# Advancements in diagnostic and interventional radiology for stroke treatment: the path from trial to bedside through the pre-MR CLEAN, MR CLEAN, and MR CLEAN II eras

**DOI:** 10.1186/s13244-023-01597-1

**Published:** 2024-01-30

**Authors:** Noor Samuels, Rob A. van de Graaf, Yvo B. W. M. Roos, Diederik Dippel, Aad van der Lugt, the MR CLEAN and CONTRAST investigators

**Affiliations:** 1 Department of Radiology and Nuclear Medicine, Erasmus University Medical Center Rotterdam, Rotterdam, the Netherlands; 2 Department of Neurology, Amsterdam University Medical Center, Location AMC, Amsterdam, the Netherlands; 3 Department of Neurology, Erasmus University Medical Center Rotterdam, Rotterdam, the Netherlands

**Keywords:** Stroke, Ischemic stroke, Neuroimaging, Endovascular procedures, Clinical trial

## Abstract

The stroke field is inevitably connected with imaging in which radiologists fulfill a central role. Our landmark MR CLEAN trial led to the implementation of baseline computed tomography angiography or magnetic resonance angiography in the acute stroke workup and subsequent endovascular treatment (EVT) for ischemic stroke patients with a large vessel occlusion in the anterior circulation, resulting in numerous patients worldwide currently being treated often successfully. A reversal of the pathophysiologic process behind an acute cerebrovascular event was made possible. Subsequently, in the MR CLEAN II trials, the clinical impact of both diagnostic and interventional radiologists remained a cornerstone of our research, which means value-based radiology. Within these MR CLEAN II trials, we proved that aspirin and heparin during EVT should be avoided due to increased symptomatic intracranial hemorrhage risk (MR CLEAN-MED). We concluded there is additional benefit of EVT in the 6-to-24-h window after stroke in the presence of good collaterals on baseline CTA (MR CLEAN-LATE). The impactful success of our stroke trials that changed many guidelines was mainly attributable to (1) the societal burden of the disease, with two thirds of patients dying or being independent at 3 months; (2) the fact that stroke is a common disease, (3) the relatively simple and pragmatic approach of the trials resembling real-world setting; (4) the acceleration of implementation in clinical practice facilitated by a structured approach to guideline development and conditional funding; and foremost (5) the excellent collaboration on a professional level between-disciplines, i.e., diagnostic radiologists, interventionalists, and neurologists.

**Critical relevance statement** The MR CLEAN and MR CLEAN II trials have had tremendous impact on clinical practice, directly by more patients being treated with an effective intervention and indirectly through adoption of evidence-based guidelines. It is in this setting of stroke treatment that diagnostic and interventional radiologists have played a crucial role and created clinical impact.

## Key points

• Endovascular treatment for stroke caused by a large vessel occlusion in the anterior circulation reduces disability significantly.

• Our landmark MR CLEAN trial led to the implementation in daily practice of computed tomography angiography or magnetic resonance angiography in the acute stroke workup and subsequent endovascular treatment.

• The key to a successful trial and fast implementation in clinical practice was the excellent collaboration between disciplines, i.e., radiologists and neurologists, in all stroke centers in the Netherlands.

• Performance of a multicenter trial, multidisciplinary guideline adaptation recommending endovascular treatment, and reimbursement were important steps resulting in treatment of 8% of all ischemic stroke patients annually.

## Introduction

Imaging has always been crucial in the diagnosis and treatment of stroke, and it requires a certain interaction between neurologist and radiologist for optimal patient care. As stroke treatment was immensely changed by our landmark MR CLEAN (Multicenter Randomized Clinical Trial of Endovascular Treatment for Acute Ischemic Stroke in the Netherlands) trial, we will focus on the clinical impact of created by clinical research on acute stroke treatment performed by diagnostic and interventional radiologists.

### Pre-MR CLEAN

The need for radiologic assessment in stroke workup starts already in the emergency room as, after a brief evaluation by the neurologist, a non-contrast computed tomography (NCCT) is required to make the crucial differentiation between hemorrhagic and ischemic stroke. This differentiation is important as it will guide the treatment decision either to administer fibrinolytic agents or to withhold this treatment. Prior to the introduction of thrombectomy treatment for large vessel occlusions in the anterior circulation, intravenous fibrinolytic agents were the main stay of acute stroke treatment for many years [1].

The problem we then encountered was that fibrinolytic agents contained well-recognized limitations. This mainly concerned the narrow therapeutic time window and the risk of intracranial hemorrhage [2]. Moreover, those fibrinolytic agents appeared to be much less effective at opening proximal large vessel occlusions, which are present in about one third of cases of acute anterior circulation stroke, in the Netherlands accounting for about 2400 patients annually [3]. In parallel to the progress being made in the field of fibrinolytic agents, there had been vast progress in the field of interventional neuroradiology with better tools and possibilities to access intracranial vasculature. It seemed an obvious next step to try to open the intracranial vessels using these devices.

From previous trials, we learned that clinical methods to detect large vessel occlusion before EVT were not sufficient to select patients for this treatment. This implied that we needed dedicated baseline computed tomography angiography (CTA) assessment in all stroke patients [4–6]. Also, we learned from these studies that intra-arterial delivery of thrombolytic agents and the first generation thrombectomy devices were not that effective in removing the intracranial occlusion with more potential for the newer generation devices of mechanical clot retrieval [4–7].

### MR CLEAN

With the decades of experience of the neurologists with trial performance in stroke and the need for imaging work-up and EVT by radiologists, a collaboration between the disciplines was the best next step to effectively prove the benefit of EVT. At this point in time, both radiologists and neurologists from different hospitals joined forces and designed the MR CLEAN trial [8, 9]. As part of this trial, we implemented CTA in the regular stroke work-up in participating centers and proved that for patients with acute ischemic stroke caused by a proximal intracranial occlusion of the anterior circulation, mechanical thrombectomy administered within 6 h after stroke onset was effective and safe. Subsequently, the results of our MR CLEAN trial had great implications for both patients and the radiological community.

The results of this trial led to the implementation of CTA along NCCT in the acute work-up of all suspected stroke patients. The crucial role of interventionalists in the treatment of ischemic stroke patients by means of EVT resulted in enforcement of the position of interventional (neuro)radiology. Many hospitals were forced to enlarge the number of interventionalists capable of performing this high-end procedure.

MR CLEAN demonstrated that it is possible to achieve functional recovery in about 15 to 25% of patients treated within 6 h with EVT. This evidence resulted in a tremendous change in the management of stroke. The instant effects on the adaption of national European and American guidelines followed by a quick implementation resulted in numerous patients being effectively treated nowadays.

### MR CLEAN II trials

After MR CLEAN ended and before the start of new interventional stroke studies, which we bundled in MR CLEAN II, we evaluated the nationwide implementation of EVT. This was done by keeping track of all patients treated with EVT in routine clinical practice in the Netherlands. This registry, containing all clinical and imaging data, was called the MR CLEAN Registry [10, 11]. This MR CLEAN registry demonstrated that in routine clinical practice, EVT for patients with acute ischemic stroke is at least as effective and safe as in the setting of a randomized controlled trial [11].

The goal then in the MR CLEAN II trials was to improve outcomes of stroke patients further following EVT. All trials in MR CLEAN II required again a crucial role for radiologist and interventionalists due to expansion of indication for EVT based on imaging selection, additional medical treatment during EVT, and imaging outcome assessment. With study outcomes recently being published, we expect that this will impact clinical guidelines once more leading to better outcomes after EVT.

## Study design

### Roadmap

The lack of evidence in multidisciplinary guidelines around 2010 (when MR CLEAN started) to treat stroke patients caused by a large vessel occlusion was the stimulus to set up the MR CLEAN collaboration. As in the Netherlands centers are relatively small in terms of individual center numbers in comparison to other countries, collaboration between centers was required. This first collaboration was set by three Dutch academic centers (Erasmus MC University Medical Center, Rotterdam; Academic Medical Center Amsterdam and Maastricht University Medical Center) in which neurologists, radiologists, and interventionalists joined forces. These early trialists designed the MR CLEAN trial and formed the basis for the latter MR CLEAN collaboration. In this collaboration, information on treated patients before the start of the trial and used techniques was shared resulting in an acceleration in knowledge acquisition on device usage and its feasibility [12]. At the start of the MR CLEAN trial, the collaboration extended quick to all 16 centers in the Netherlands that were able to provide EVT. The collaboration once set in the MR CLEAN trial was, to our knowledge, the first nationwide Dutch trial ever performed. This collaboration was subsequently continued in the MR CLEAN Registry and in the MR CLEAN II trials.

### Assembling the study team

**Fig. 1:**
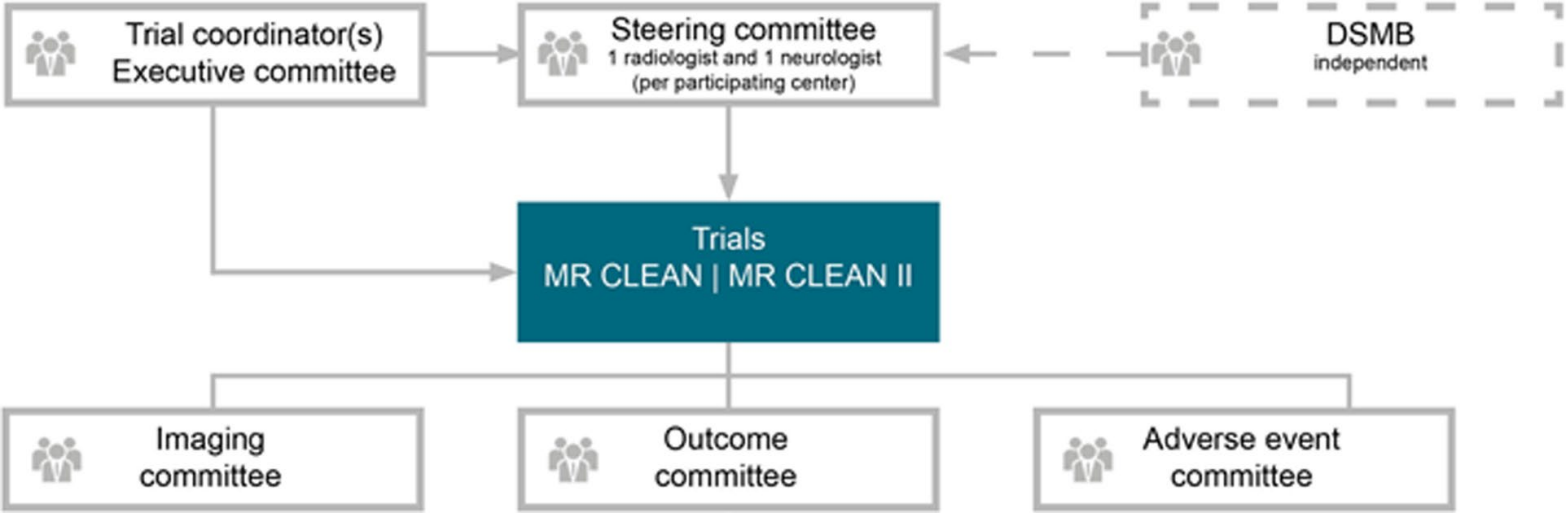
Trial organization

At the start of the MR CLEAN collaboration, it was decided that EVT centers could only participate when they were willing to be represented by two local principal investigators (PIs), i.e., a stroke neurologist and (neuro-)interventional radiologist, in the steering committee. This requirement stimulated the collaboration locally in the participating EVT centers by giving both disciplines an equal vote in the local hospital management and the national trial management. Over time, the general core structure with regard to the study team remained the same (Fig. 1). In this structure, the trial steering committee is the main decision-making body (e.g., protocol changes, continuation of the trial). It consists of local PIs, a stroke neurologist and a (neuro-)interventionalist from each participating center, the members of the executive committee, and the trial statistician. The trial executive committee consists of a team of around six principal investigators, one or more coordinating junior researchers (post-docs or PhD students), and the trial statistician and prepares documents for the steering committee. The trial executive committee also forms the writing committee for the trial. The study coordinators are responsible for running the trial on a day-to-day basis and report to the executive committee. Other important committees are the imaging assessment committee, the outcome committee, and the serious adverse event committee. More information on specific trial design can be found in the trial protocols [9, 13, 14].

### Funding

The MR CLEAN was executed with funding from the Dutch Heart Foundation and several small grants from industry. During the conduct of MR CLEAN, the health care system in the Netherlands reimbursed EVT for ischemic stroke only when patients were included in the trial. This policy resulted in high recruitment rates and avoided the “cherry picking” of presumably easy-to-treat patients, which were key factors to success.

After the finalization of the MR CLEAN trial, the collaboration between the centers was formalized in the CONTRAST (Collaboration for New Treatments of Acute Stroke) consortium which executed the MR CLEAN II trials with funding from the Dutch Heart Foundation and several large grants from industry.

## Reflection on methodology and approach

We aimed with our trials, the MR CLEAN trial and subsequent MR CLEAN II trials, to maintain pragmatic designs. This means that we adopted a relatively simple approach trying to resemble as close as possible the real-world clinical setting. For example, several thrombolytic agents and all commercially available mechanical devices with some evidence on efficacy and CE marking were allowed. Also, the broad patient selection criteria in the trials were key features of the pragmatic design. This design made broad implementation of trial results into clinical practice relatively easy.

## Implementation and impact

### MR CLEAN

The success with functional recovery achieved in about 15 to 25% of patients treated within 6 h with EVT, more than in controls, led to huge change in the management of stroke. Instantly, the course of other trials evaluating effect of EVT was affected such as EXTEND IA, SWIFT–PRIME, ESCAPE, REVASCAT, THERAPY, THRACE, and PISTE, leading to immediate suspension of most of these studies [15–21]. Quickly—in terms of months—after the result came out, EVT became implemented in national and international guidelines [22, 23]. In the Netherlands, the efforts of the national professional society of radiologists (Nederlandse Vereniging voor Radiologie, NVVR) and neurologists (Nederlandse Vereniging voor Neurologie, NVVN) worked together on the guideline facilitated a fast implementation of EVT in clinical practice. In addition, criteria for primary stroke center and EVT center as well as training requirements for interventionalist were updated or newly formulated and implemented. Also, the early involvement of the health insurance companies, while patients were still included in MR CLEAN, is making them aware of the study and the potential results, and discussing the reimbursement of EVT in an early stage turned out to be a good strategy. It enabled the EVT centers to continue with their EVT program and facilitated a fast nationwide implementation of EVT after the trial ended.

**Fig. 2:**
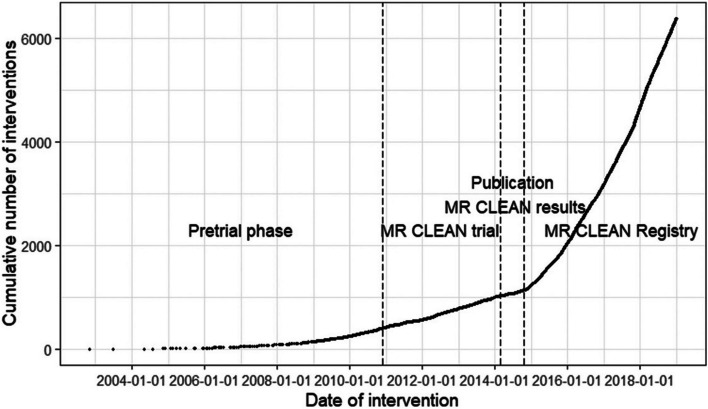
By courtesy of Wiegers et al. showing the cumulative number of patients being treated with endovascular treatment in the Netherlands between 2004 and 2019

The quick implementation in clinical practice of EVT resulted in an increase in patients being treated, probably caused by an increase in awareness among the primary stroke centers. In the Netherlands, this resulted in 3294 patients being treated with EVT between April 2014 and October 2017, which at that time was an exponential increase (Fig. 2) [24]. The same trend was seen in other countries. In the USA, case volumes doubled in all centers from 2013 to 2016 at EVT-capable hospitals. This accounted for 27.1% patients treated with EVT in Q3 of 2016 of the potentially eligible patients (based on time of presentation and stroke severity) at EVT-capable hospitals (491,879 patients potentially eligible for EVT from 448 hospitals between 2003 and 2016) [25]. Since then, the number of patients being treated with EVT, both due to awareness and extension of inclusion criteria, kept rising.

## MR CLEAN II

We expect that the MR CLEAN II trial results again will impact the stroke guidelines. For example, in MR CLEAN MED, we found that aspirin and heparin administration during thrombectomy should be avoided. This will impact the guidelines as previous studies indicated that heparin administration is heterogenous among centers [26]. In MR CLEAN LATE, we demonstrated that EVT was efficacious and safe for patients in the 6-to-24-h window selected on the presence of collateral flow on CTA [27]. Again, this will impact clinical guidelines as well and will lead to more patients referred for EVT. Lastly, in MR CLEAN NOIV, we demonstrated that EVT alone was neither superior nor non-inferior to intravenous alteplase followed by EVT with regard to disability which could make work-up easier for some patients directly referred to EVT centers [28].

## Lessons learned

### What worked well

The way the collaboration between centers and within centers was organized works well. It started with the MR CLEAN trial and then continued during the MR CLEAN Registry (observational cohort of patients containing clinical and imaging data) and formalized in the CONTRAST consortium. It created a collaboration in which expertise divided over several disciplines were combined to perform ground-breaking clinical research. Each participant could contribute and receive return on investment as the results were directly implemented in their own institution.

What also worked well was the creation of a *semi-open* database which could be accessed by all collaborators following an application to the trial committee requesting data for a specific sub-study either on the MR CLEAN trial data, MR CLEAN Registry data, or currently on the MR CLEAN II data. The formed team particularly interested in the sub-study and fulfilling the ICMJE criteria of co-authorship published the paper on behalf of all collaborators [29]. This scientific output was another incentive for investigators to be involved in the collaboration. Timely observational analyses of the MR CLEAN trial and MR CLEAN Registry led to new hypotheses that could be tested in the subsequent MR CLEAN II trials (e.g., for MR CLEAN NOIV [30], MRCLEAN MED [26, 31], and MR CLEAN LATE [32–35]).

### Challenges

Specifically, for the MR CLEAN trial, the most difficult practicality in the starting phase was to get an interventional team up and running 24/7 in EVT capable centers. During the trial, trialists realized that, inevitably, they were part of a wave of progress in stroke care. This increased their enthusiasm and efforts in contributing to the trials.

The restricted amount of money available for a trial of this size is in hindsight ridiculous in comparison to the funds for industry driven studies. In this setting, the reimbursement for EVT was highly effective in the acceleration of this trial; however, this policy is not always possible in clinical research. Yet, imagine what progress the medical device field would encounter if this strategy was the rule [36].

One of the difficulties of the MR CLEAN II trials was the simultaneous start of multiple stroke trials. For the smaller centers, it was sometimes difficult to get all the field work done (e.g., a lot of paper work). However, the intense collaboration between the individual EVT centers and the trial coordinators sharing experience was key to success.

One pitfall in a collaboration like ours, in which a huge amount of data was collected, is the temptation to compare the technical and clinical results of EVT centers and individuals. However, the case-mix at an individual center makes straightforward conclusions on center quality difficult and dangerous [37]. To avoid this and to still keep track of individual center performances in such a collaboration, centers could receive individual benchmark feedback comparing their data to the *masked* data of the others. In this way, it was still possible to discuss individual benchmark parameters without putting a collaboration at stake.

## Conclusion

The MR CLEAN and MR CLEAN II trials have had tremendous impact on clinical practice, directly by more patients being treated with an effective intervention and indirectly through adoption of evidence-based guidelines. This was why the former editor in chief of the New England Medical Journal mentioned the MR CLEAN trial report as “the most practice-changing and lifesaving paper from the past 19 years” [36]. It is in this setting of stroke treatment that diagnostic and interventional radiologists have played a crucial role and created clinical impact.

There were some main reasons for the methodological success of our research. First, the severity of the condition. Before the introduction of mechanical thrombectomy, two thirds of patients died or became functional independent. With the introduction of mechanical thrombectomy, a *reversal of the pathophysiological process* of cerebral ischemia was made possible. Second, stroke caused by a large vessel occlusion in the anterior circulation is a common disease. Currently, in the Netherlands, about 2400 EVT eligible patients present within 6 h annually, and indications and time window for treatment are extending [3]. Third, the trial had a relatively simple and pragmatic approach resembling the real-world setting, making the results of the trial easy to implement in clinical practice. Fourth, the implementation of EVT in clinical practice was accelerated by the structured approach to post-trial guideline development and the reimbursement policy during and after the trial of the insurance companies. Fifth, the excellent well-organized collaboration between disciplines, i.e., diagnostic radiologists, interventionalists, and neurologists, and on a national level between stroke centers with input from all collaborators were key elements to achieve a change in stroke treatment.

## Data Availability

Not applicable, as no data was needed for the conduct of this study.
